# Medication adherence, lifestyle practices and blood pressure control in hypertensive patients: A questionnaire-based analysis

**DOI:** 10.6026/973206300221924

**Published:** 2026-04-30

**Authors:** Kumaran Ottilingam Ravindran, Saifudheen Muhammeds Ali, Shilpa Hajare

**Affiliations:** 1 Department of Internal Medicine, Madras Medical College, EVR Periyar Salai, Park Town, Chennai, Tamil Nadu, India; 2 Department of Medicine, University hospitals of Dorset, Poole, England, United Kingdom; 3 Department of Community Medicine, N.K.P. Salve Institute of Medical Sciences & Research Centre and Lata Mangeshkar Hospital, Nagpur, India

**Keywords:** Hypertension, medication adherence, knowledge-attitude-practice (KAP) survey

## Abstract

Uncontrolled hypertension is one critical contributing factor to cardiovascular disease and continues to cause problems even through there are effective pharmacological and lifestyle interventions available. In this cross-sectional study, researchers collected data from 150 hypertensive outpatients on their medication adherence, lifestyle practices as well as how these factors related to their ability to control blood pressure using a structured knowledge attitude and practice questionnaire and clinical assessments. The systolic blood pressure average for these subjects was 142.5 ± 13.9 mmHg, while diastolic blood pressure was 88.7 ± 9.6 mmHg; therefore only 60% were able to attain appropriate blood pressure control. There was a statistically significant correlation between KAP and adherence scores and lower systolic (r = -0.38) and diastolic (r = -0.34) blood pressure (p < 0.05). Those patients who had optimal practice for lifestyle modification achieved the greatest percentage of individuals that attained blood pressure control (82%). Thus, enhance adherence and lifestyle interventions through structured education and behavioural support will help control hypertension.

## Background:

Worldwide, hypertension is a major modifiable risk factor for cardiovascular disorders, including stroke and chronic kidney disease (CKD) [1]. Presently, it is projected that greater than one billion individuals are impacted by hypertension, with a large percentage failing to have their BP controlled, even though there are effective treatments available to them [1, 2]. To effectively manage hypertension, individuals will require a sustained effort to adhere to prescribed medications, as well as changes to their lifestyle through a reduction in dietary salt consumption, an increase in regular physical activity, controlling body weight and managing stress [3, 4]. Unfortunately, poor adherence to medications, as well as sub-optimal lifestyle practices remains among the most significant barriers to reaching blood pressure target goals for hypertensive individuals [5]. A person's knowledge, attitudes and daily practices play a large role in their ability to adhere to their prescribed medications and maintain long-term control of their hypertension. Recent studies have shown that hypertensive patients who do not fully understand hypertension; have misconceptions about chronic therapy; and do not engage in adequate behavioural practices fail to control their BP [6 7-8].

Pharmacologic strategies for managing hypertension have been shown to be effective; however, the behavioural determinants of adherence to pharmacologic therapy and compliance with lifestyle modifications limit the effectiveness of hypertension therapy in real-world practice [9]. In order to identify modifiable factors impacting a person's BP levels, structured KAP assessments can be employed to systematically assess the behavioural determinants of adherence and lifestyle modification [10]. Therefore, it is of interest to examine the relationship between medication adherence and lifestyle modification among those diagnosed with hypertension, to determine association between behaviours and BP control.

## Materials and Methods:

Participants in this study consisted of 150 adults (ages 30-75) who were diagnosed with essential hypertension and visited an outpatient tertiary care facility between January - June 2024. To qualify, the patients must have been prescribed antihypertensive medications for at least six (6) months. Patients who had secondary hypertension, advanced heart failure, chronic renal failure, or cognitive deficit were excluded. All patients provided written informed consent and ethical approval to conduct the study was granted by the Institutional Review Board. The study was conducted using a pre-tested structured KAP (knowledge, attitude and practice) questionnaire, which was made up of thirty (30) questions categorized into three distinct categories. In the knowledge section, participants were asked about their general awareness of hypertension, its possible complications and what medications they take. In the Attitude section, the participants answered questions about their beliefs concerning the need for long-term therapy and their motivation to maintain a healthy blood pressure level. In the Practice section, participants were asked questions asked about how well they adhered to their medication regimen(s), made dietary changes, exerted themselves physically, managed stress and returned for follow-up appointments. Once participants completed the KAP questionnaire, the average score from the three KAP categories was calculated and then placed into one of three categories; good, average, or poor. Adherence to prescribed medications was measured using a modified morisky adherence scale. Blood pressure readings were obtained with the use of a calibrated sphygmomanometer. Blood pressures were taken two times and two minutes apart after the patient had a five- minute resting period and the average of both readings recorded as the patient's blood pressure reading. According to the JNC 8 guidelines, blood pressure control was defined as less than 140/90 mmHg. Sociodemographic data points collected were age, sex, length of time the patient had been hypertensive and type and number of medications taken. Data were analyzed by means of SPSS version 26. Descriptive statistics were used to report categorical variables as percentages and frequency counts and continuous variables as means ± standard deviations. Pearson correlation was used to examine the relationship between total KAP scores and systolic and diastolic blood pressures. The statistical significance level used was p < 0.05.

## Results:

A total of 150 hypertensive patients were included in the analysis. The cohort comprised 56% males and 44% females, with a mean age of 56.2 ± 9.8 years. The mean duration of hypertension was 8.1 ± 4.5 years. The mean systolic blood pressure was 142.5 ± 13.9 mmHg and mean diastolic blood pressure was 88.7 ± 9.6 mmHg. Overall, 60% of participants achieved blood pressure control. The mean total KAP score was 17.6 ± 4.3, indicating moderate awareness. Good knowledge was observed in 42% of participants, positive attitude in 48% and optimal practice in 46%. Medication adherence was reported by 63%, while lifestyle practices such as salt restriction, regular exercise and stress control were less frequently adopted. Blood pressure control varied significantly across knowledge, attitude and practice categories. Higher total KAP scores were negatively correlated with systolic and diastolic blood pressure (p < 0.05). Table 1 (see PDF) shows that 56% of participants were male and 44% were female, with a mean age of 56.2 ± 9.8 years and mean hypertension duration of 8.1 ± 4.5 years, while the mean systolic and diastolic blood pressures were 142.5 ± 13.9 mmHg and 88.7 ± 9.6 mmHg respectively and 60% achieved blood pressure control. Table 2 (see PDF) indicates that 42% of participants demonstrated good knowledge of hypertension, 40% had average knowledge and 18% had poor knowledge, reflecting variability in disease awareness. Table 3 (see PDF) demonstrates that 48% of participants exhibited a positive attitude toward hypertension management, 35% maintained a neutral attitude and 17% expressed a negative attitude toward long-term therapy and lifestyle modification. Table 4 (see PDF) shows that 63% reported medication adherence, whereas adherence to lifestyle measures was lower, with 54% practicing salt restriction, 49% engaging in regular exercise and 46% adopting stress control strategies. Table 5 (see PDF) compares blood pressure control across knowledge categories and shows that 78% of participants with good knowledge achieved control compared to 55% with average knowledge and 34% with poor knowledge. Table 6 (see PDF) depicts that 80% of participants with a positive attitude achieved blood pressure control compared to 54% with neutral attitude and 32% with negative attitude. Table 7 (see PDF) highlights that 82% of participants with optimal practice achieved blood pressure control, whereas control declined to 60% in moderate practice and 36% in poor practice categories. Table 8 (see PDF) demonstrates a significant negative correlation between total KAP score and systolic blood pressure (r = -0.38) as well as diastolic blood pressure (r = -0.34), with statistical significance (p < 0.05), indicating that higher KAP scores were associated with lower blood pressure values.

## Discussion:

This research evaluated the relationship between patient adherence to prescribed medications and lifestyles and the overall control of a hypertensive population's blood pressure levels. Results of the study show moderate overall knowledge, attitude and practice (KAP) scores in this population; however, KAP scores were found to be very diverse regarding knowledge, attitude and practice [11]. While 60% of participants achieved their target blood pressure levels, control rates were much lower for each behavioural category considered. Higher KAP scores were found to be statistically significantly related to lower systolic and diastolic blood pressure values. These data support the belief that behavioural determinants of hypertension management are important clinically [12]. There were identified knowledge deficits, as only 42% of participants were able to demonstrate a good understanding of hypertension. A noted relationship was found between the levels of blood pressure control and levels of knowledge; the percentage of participants with controlled blood pressure who had good knowledge was 78% compared to only 34% of the participants in the poor knowledge category [13]. Similar results have been reported in many recent studies that suggest that disease-specific education leads to better patient compliance and clinical outcomes. Additionally, adequate knowledge assists in the recognition of potential complications and helps to reinforce adherence to long-term therapy [14]. Attitude was also found to have a strong relationship to blood pressure control. Of those participants with a positive attitude, 80% achieved blood pressure control, while those who reported having a negative attitude showed significantly lower rates of control. Attitude also affects an individual's level of motivation, persistence in therapy and participation in behavioural change [15]. The behavioural models established in managing chronic diseases point to the fact that the attitudes individuals develop regarding their necessity for treatment and their perceived benefits from the treatments dramatically influence the behaviour of that individual to adhere to therapy [16]. Of the three behaviour-related variables evaluated in this study, practice was found to be more related to the use of approved pharmacotherapy than the other two variables. For example, 63% of participants reported adhering to their prescribed medications, whereas fewer participants reported consistently engaging in the necessary lifestyle changes (e.g., salt restriction, exercise, stress control). This situation indicates the continued existence of the knowledge-practice gap, which is commonly found in hypertension management. This implies that although pharmacotherapy may be prescribed, failure to implement appropriate lifestyle changes hinders the individual from achieving optimal control over their blood pressure [17, 18]. The current study's negative relationship between total KAP scores and both systolic and diastolic blood pressure provides a quantification of the relationship between behavioural variables and clinical disease. Higher total scores were related to lower blood pressure values; in essence, the combination of knowledge, positive attitudes and regular practice means synergistically to provide better control. These findings are in agreement with recent information that has shown that implementing both educational and behavioural components in an integrated manner reduces cardiovascular risk as well as blood pressure levels [19]. Significantly, the current study adds to the literature by correlating structured KAP variables to objective blood pressure measurements taken at a routine outpatient setting. Historically, most of the studies published have evaluated adherence/increase in lifestyle individually. However, the current study demonstrates a gradient when comparing KAP scores across all behavioural variables; this demonstrates the cumulative influence of these behavioural variables on hypertension outcomes [20]. Several limitations in conducting the current study should be noted. The cross-sectional design prevents establishing any causality. Furthermore, because data regarding lifestyle changes were self-reported, there is a potential for recall bias. Additionally, as the study was conducted at a single institution, generalizability is limited. Therefore, the authors suggest that longitudinal studies assess the impact of disease specific interventions directed at improving KAP and the subsequent changes in blood pressure over time. In summary, the results of the current study support the value of a multidimensional approach for managing hypertension that includes pharmacological treatment, structured education, behavioural interventions and encouragement to make lifestyle changes. There is a clear need for strategies that increase patient-centred adherence to achieve optimal control of blood pressure and minimise the risk of long-term complications associated with cardiovascular disease.

## Conclusion:

Higher medication adherence and consistent lifestyle modification are significantly associated with better blood pressure control among hypertensive patients. Graded improvements across knowledge, attitude and practice domains corresponded with progressively higher control rates and lower blood pressure values. Thus, integrating structured KAP-based education and behavioral reinforcement into routine hypertension care may substantially reduce the burden of uncontrolled blood pressure and cardiovascular risk.

## Acknowledgement:

We acknowledge that the first and second author contributed equally to this paper and hence they are considered as joint first author

## References

[1] Villafuerte FU (2020). Medicine (Baltimore)..

[2] Ito M (2024). BMC Prim Care..

[3] Odunaye-Badmus SO (2024). West Afr J Med..

[4] Ahmed EHH (2025). S Afr Fam Pract (2004)..

[5] Gömleksiz M (2025). J Clin Hypertens (Greenwich)..

[6] Dukunde A (2023). Diseases..

[7] Mills KT (2020). Nat Rev Nephrol..

[8] Kebede T (2022). PLoS One..

[9] Joudeh A (2023). Medicine..

[10] Setiadi AP (2022). PeerJ..

[11] Xu L (2024). J Clin Hypertens..

[12] Waris B (2025). Cureus..

[13] Farhadi F (2023). BMC Cardiovasc Disord..

[14] Trefond J (2022). BMC Prim Care..

[15] Ahmed A (2024). ARYA Atheroscler..

[16] Bogale S (2020). SAGE Open Med..

[17] Dhakal A (2022). J Clin Nurs..

[18] ALruwaili BF (2024). Medicina..

[19] Zhang Z (2025). Sci Rep..

[20] Ouaddouh Y (2025). Curr Hypertens Rev..

